# Supplementary material for: Are carbon emissions trading and green financial instruments synergistic? -Comprehensive quantitative research based on content analysis
Source: PLoS One. 2024 Mar 7;19(3):e0298601. doi: 10.1371/journal.pone.0298601 (PMC10919601; doi:10.1371/journal.pone.0298601)
Supplement: S1 Data — (ZIP) [file pone.0298601.s001.zip › Data/地级市Z_F工作报告.zip!/▒▒╛⌐/└┤╫╘╣1⁄2╓┌║┼í╛┬φ┐╦╩2╛▌═°í┐_2005.docx]

　　各位代表：

　　现在，我代表北京市人民政府，向大会作政府工作报告，请予审议，并请市政协各位委员提出意见。

　　一、2004年工作的回顾

　　过去的一年，在中共北京市委的领导下，首都人民坚持以邓小平理论和“三个代表”重要思想为指导，认真贯彻党的十六大和十六届三中、四中全会精神，以及中央对北京市工作的一系列重要指示，树立和落实科学发展观，推进实施“新北京、新奥运”战略构想，首都改革开放和现代化建设又迈出了新的步伐。市十二届人大二次会议确定的各项目标基本实现。

　　——首都经济平稳较快发展。北京市生产总值达到4283.3亿元，比上年增长13.2%，是近十年来经济增长最快的一年。地方财政收入744.5亿元，增长29.7%。居民消费价格指数为101%。

　　——人民生活水平稳步提高。城镇居民人均可支配收入15638元，实际增长11.5%；农民人均纯收入7172元，实际增长9.2%。城镇登记失业率1.3%。人口自然增长率为0.7‰。

　　——环境质量进一步改善。空气质量实现了控制目标，达到62.5%；城八区污水处理率达到58%，生活垃圾无害化处理率达到93.8%；城八区绿化覆盖率达到41.8%，全市林木覆盖率达到49.5%。一年来，我们按照科学发展观的要求，转变发展思路，调整工作部署，注重统筹协调发展，注重结构调整与优化，注重增长方式的转变，注重强化城市管理，切实加强经济社会的薄弱环节，主要做了以下几个方面的工作：

　　高度重视“三农”问题，切实保护农民利益。大幅增加对郊区的投入，用于基础设施建设的政府投资达到26.2亿元，增长1.4倍；用于社会事业的财政资金20.5亿元，增长1.1倍。深化农村改革，免征农业税及其附加，基本完成农村土地确权工作，修订建设征地补偿安置办法，妥善解决拖欠农民征地补偿款、建设征地转工自谋职业人员参加社会保险等历史遗留问题。加大对农民的直接补贴，落实粮食生产直补政策，建立山区生态林补偿机制，对乡镇和村级组织给予直接补贴和补助，提高城市绿化隔离地区、绿色通道生态林建设占地补偿和养护补助标准，对防控禽流感期间禽类被扑杀受损失的农户给予了直接补助。重新启动山区搬迁工程。216个村铺通了柏油路。2004年是近年来制定实施“三农”政策最多、力度最大、农民得到实惠最多的一年。

　　坚决贯彻落实中央宏观调控政策措施，促进经济增长速度与质量、效益相统一。各类开发区由470个减少到28个，撤销94%，核减规划面积4.7万公顷。依法清理在建和拟建项目，规范土地出让方式，处理了一批历史遗留问题。固定资产投资和信贷投放得到有效控制。加强经济运行调节，保持了煤电油运供需基本平衡，确保了粮食等重要商品的市场供应。加快高新技术产业和现代制造业发展，工业投资大幅增加，多点支撑的增长局面初步形成，工业增速达到近年来最高水平。现代服务业增势良好，文化产业初具规模，会展经济稳步增长。实际利用外商直接投资30.8亿美元，增长43%；地方企业出口完成106.1亿美元，增长44%。入境旅游人数创历史新高。经济运行质量和效益进一步提高，全市规模以上工业企业利润增长32%，商业企业利润增长1.1倍。

　　加大投入和调整力度，均衡发展各项社会事业。市级财政用于全市社会事业的资金达到132.8亿元，增长27.8%。加强公共卫生体系建设，城乡一体的紧急医疗救援体系建设稳步推进，乡镇卫生院和村卫生室的改造加快，农村药品供应网络得到完善。积极推进中小学布局调整，安排4.8亿元支持农村中小学信息化和办学困难学校建设。投入专项经费帮助困难家庭子女完成义务教育，基本解决来京务工人员子女义务教育问题。调整科技投入结构，支持企业科技创新与成果转化，一批科研院所转制稳步推进。文化事业繁荣发展，文化体制改革试点工作进展顺利，中法文化年北京承办活动取得成功。古都风貌得到进一步保护，“3.3亿元文物古建抢险修缮工程”完工。群众体育活动蓬勃开展，竞技体育取得可喜成绩，北京体育健儿在雅典奥运会上勇夺5枚金牌。落实人才强国战略，制定实施吸引文化、体育等领域人才的政策措施。人口和计划生育工作稳步开展。

　　统筹规划建设管理，促进城市可持续发展。按照政府组织、依法办事、专家领衔、部门合作、公众参与、科学决策的方针，创新规划编制方式，集中各方面智慧，完成了城市总体规划的修编，确定了到2020年北京城市建设和发展的规划蓝图。编制了城市环境建设规划和交通发展纲要。在加快轨道交通和城市道路建设的同时，调整存量，挖掘潜力，加强管理，取消五环路收费，打通一批断头路，改善城市道路微循环，四环路以内道路通行能力提高了15%。第一座医疗废物集中处理厂一期投入运行，卢沟桥污水处理厂和清河污水处理厂二期建成。实施控制大气污染第十阶段措施，二氧化硫年均浓度首次达到国家空气质量二级标准。完成城区绿化1000公顷，实施112项重点环境整治项目。全方位推进节约用水工作，机井装表率达到96%，压缩社会单位供水指标5400万立方米。加强社会宣传，发布致市民公开信，全社会的资源节约意识有所增强。

　　全面落实“节俭办奥运”方针，稳步推进奥运筹备工作。确立奥运场馆建设“安全、质量、功能、工期、成本”五统一原则，优化调整了比赛场馆的选址、规模、标准和规划设计方案，完成所有新建场馆设计方案的征集评审工作。成立“2008”工程建设指挥部，加大奥运工程建设组织协调力度，与31个比赛场馆业主或责任单位签订承诺书。配合奥组委成功组织雅典奥运会火炬在京传递活动，制定了人文奥运行动计划实施意见。

　　加大改革力度，优化发展环境。国有企业改革全面推进，国有资产监管进一步加强。组建了北京产权交易所。继续推进基础设施建设运营市场开放，一批项目通过特许经营实现市场化融资。开展公益性政府投资项目代建制试点，社会投资项目审批逐步放开。建立全市集中统一的水资源管理体制，推进国土资源垂直统一管理，完成区县政府机构改革。全面实施《行政许可法》，本市设定的行政许可事项精简59.3%，行政性收费项目取消14项。深化政务公开，大力推广全程办事代理制和“一站式”办公，成立北京市行政投诉中心。进一步清理和精简企业审批、收费、年检事项。降低企业准入门槛，新增企业数量创历史新高。加强知识产权保护，深入开展专项整治，初步建立了食品安全监管体系，整顿规范市场经济秩序取得明显进展。

　　认真做好就业和社会保障工作，着力改善人民生活。56件实事全面落实。完善促进就业政策，将有劳动能力的农转居人员、残疾人员和城镇低保人员纳入失业登记和就业服务范围，新增城镇就业23.4万人。加大帮扶力度，5.3万“4050”失业人员实现再就业，5.5万农村富余劳动力实现转移就业。健全社会保障体系，23.6万名城乡低收入人员纳入最低生活保障范围，实现了应保尽保。实施城市低保分类救助政策，上浮了特困人员救助标准。扩大整建制农转居人员参加社会保险试点，确立了农村养老保险制度模式和筹资方式，新型农村合作医疗覆盖面达到64%。提高了城镇基本医疗保险的保障水平，实施了来京务工人员参加工伤和医疗保险办法。危旧房改造在调整中继续推进，拆除危房近30万平方米，动迁居民2.4万户。基本完成标准租私房承租户腾退工作。改善城乡居民饮用水水质，解决19万人安全饮水问题。改造、新建了400座标准公共厕所，考虑到洗浴水价调整给一部分低收入群众带来的困难，在市区布局建设了57家便民低价浴池。

　　强化城市安全工作，维护首都安全稳定。打赢了防控高致病性禽流感和非典疫情阻击战，保障了人民生命健康安全。深入排查多年积累的生产安全和社会公共安全隐患910项，消除828项，尚未消除的正在加快整改。明确了政府部门安全监管职责，逐级落实安全责任制和责任追究制。建立了全市统一的应急指挥系统，制定实施《北京市突发公共事件总体应急预案》以及各类专项应急预案，加强各类专业化应急队伍建设，防灾减灾能力显著增强。认真开展人民内部矛盾纠纷排查调处工作，积极化解集体访及各类重点社会矛盾，切实维护人民群众合法权益。依法严惩各种违法犯罪行为，强化社会治安综合治理，加大反恐防暴工作力度，严密防范、坚决打击境内外各类敌对势力的图谋和破坏活动。民兵、预备役部队建设和征兵工作进一步加强。

　　切实搞好精神文明建设和民主法制建设。按照中央和市委的统一部署，狠抓了“三个代表”重要思想和党的十六届三中、四中全会精神的学习教育活动，社会主义精神文明建设取得新的进步。在全社会广泛征集新童谣，创造了加强未成年人思想道德建设的新方式。廉政建设和反腐败斗争深入开展。普法工作得到加强。民族、宗教、侨务和对台工作稳步发展。对外交往与合作更加广泛。国防教育、双拥共建继续推进，军政、军民团结进一步加强。更加重视妇女儿童和老龄工作。通过无障碍示范城市验收，残疾人事业又有新的发展。

　　认真执行市人大及其常委会的决议，坚持重大事项向市人大常委会报告制度和向市政协通报制度。市政府一名副市长和3名部门负责人向人大常委会述职。办复全国和市人大代表议案和建议1841件、政协委员提案1422件，全面有力地推动了政府工作。全年提请市人大常委会审议通过地方性法规12项，颁布政府规章14项。

　　各位代表，一年来首都改革开放和现代化建设所取得的成绩，离不开党中央、国务院的正确领导，离不开各个方面的关心、支持和帮助，是全市人民共同奋斗的结果。在此，我代表市政府，向在各个领域和岗位上辛勤劳动、做出贡献的全市人民，向给予我们支持与监督的人大代表、政协委员、各民主党派、工商联和无党派人士、各人民团体、社会各界人士，向大力支持我们工作的中央在京单位、人民解放军和武警部队以及各兄弟省、自治区、直辖市，向所有关心支持首都建设的香港特别行政区同胞、澳门特别行政区同胞、台湾同胞、海外侨胞和国际友人，表示崇高的敬意和衷心的感谢！

　　回顾过去一年，我们也清醒地看到，贯彻落实科学发展观刚刚起步，实现“五个统筹”在认识上需要不断深化，在实践中需要不断探索；与举办有特色、高水平奥运会和各项工作走在全国前列的要求相比，不少方面还存在相当大的差距。一是水、土地和能源等资源约束进一步加剧，资源利用效率不高，转变增长方式刻不容缓。二是经济体制改革相对滞后，发展的软环境差距较大，招商引资中的遗留问题迫切需要解决；社会事业资源整合、布局调整进展缓慢。三是随着城市化进程加快，城市管理难度加大，重建设、轻管理的问题仍然突出，环境污染、交通拥堵和环境脏乱的治理任务艰巨，生产安全和社会公共安全形势依然严峻，统筹协调旧城区风貌保护、人口疏散和危房改造的难度大，流动人口管理工作薄弱，城市管理水平亟待提高。四是建设社会主义和谐社会面临许多挑战，就业的结构性矛盾比较突出，区域、行业、群体之间的收入差距明显，农民增收的基础不稳，低收入群体生活比较困难；城市建设发展中的征地和房屋拆迁矛盾不容忽视；一些地区和场所社会治安问题突出，侵财违法犯罪增多；群体性事件呈现上升势头。五是社会守法意识、公德意识有待增强，精神文明建设总体水平亟需进一步提高。六是政府职能转变尚未到位，基础工作薄弱，办事效率不高；极少数干部以权谋私、腐败堕落。上述问题，我们将高度重视并认真研究解决。我们真诚期望各位代表、政协委员，各民主党派、工商联和无党派人士以及广大人民群众给予批评和监督，帮助我们把政府工作搞得更好。

　　二、2005年工作的主要任务

　　2005年是实施“十五”计划的最后一年，是筹办奥运的关键之年。现在距离举办北京奥运会仅有三年多的时间，搞好各项筹办工作，加快首都改革开放和现代化建设，任务艰巨，时不我待。今年市政府工作总的要求是：以邓小平理论和“三个代表”重要思想为指导，认真贯彻党的十六大、十六届三中、四中全会和中央对北京市工作的一系列重要指示精神，全面落实市委九届九次全会部署，以科学发展观统领全局，协调城乡发展，推进体制创新，提高开放水平，调整经济结构，转变增长方式，发展首都经济，强化城市管理，构建社会主义和谐社会，推进精神文明建设和民主法制建设，为实现“新北京、新奥运”的战略构想奠定更加坚实的基础。

　　为充分体现上述工作要求，树立正确的政绩观，市政府对今年经济社会发展的预期目标设置进行了改革和调整。主要调控目标是：城镇登记失业率控制在2.3%以内，地方财政收入增长13%，居民消费价格指数控制在103%以内，万元生产总值能耗下降5%；主要预测指标是：北京市生产总值增长9%，城镇居民人均可支配收入和农民人均纯收入分别实际增长6%以上。

　　今年，要重点抓好以下几个方面的工作。

　　(一)紧紧抓住奥运机遇，全面提升城市建设管理水平

　　按照举办一届有特色、高水平奥运会的要求，全市建设管理工作必须充分体现“绿色奥运、科技奥运、人文奥运”三大理念，倒排工期，周密部署，确保各项筹备工作准时、高质量地完成。

　　全力搞好奥运场馆及配套设施建设。严格遵循“五统一”原则，按照计划安排，全面开工新建场馆和国家会议中心、奥运村、记者村等设施，适时启动改扩建项目，抓紧落实临时设施建设方案。加快场馆周边道路及市政配套设施建设，启动奥林匹克中心区广场、水系、绿地等景观工程。加强监督、审计，实施“阳光工程”，确保“廉洁办奥运”。全力配合奥组委做好各项筹备工作。

　　切实搞好城市基础设施建设。加快建设4条轨道交通线路，新建机场北线等高速公路，建设莲花池西路、通惠河北路等城市快速路，以及朝阳路等城市主干路。继续改善城市道路微循环系统，推进回龙观等8个重点地区的道路及公共客运系统建设，做好五环路内道路交通组织优化工作，完成南中轴路大容量快速公交建设。促进能源供应结构调整，重点抓好陕京天然气二线市内配套工程、公主坟和车公庄地区热力管网西延工程，发展天然气用户12万户、集中供热面积300万平方米。加快5座污水处理厂建设，完成40公里城市河段治理任务。开工建设南水北调重点控制性工程。城八区、郊区生活垃圾无害化处理率分别达到94%和40%，污水处理率分别达到70%和40%。

　　进一步加强大气污染治理和生态建设。制定实施第十一阶段控制大气污染措施，继续削减重点企业污染物排放总量，2000台燃煤锅炉改用清洁能源，淘汰3800辆老旧柴油公交车，按照欧Ⅲ排放标准更新2万辆出租车，加强工地扬尘污染监管，巩固治理成果，市区空气质量二级和好于二级的天数达到63%。继续抓好山区、平原、绿化隔离地区三道绿色生态屏障建设，加强抚育管护，全市林木覆盖率达到50%。结合城市防灾避险设施建设和节水、集水的需要，积极推进公园绿地和园林景观道路等绿化美化工程建设，城八区绿化覆盖率达到42.5%。

　　大力推进城市环境建设。认真落实《北京市城市环境建设规划》，从城市中心逐步向外围推进，确保2007年完成环境建设各项任务。今年重点整治三环路以内及奥运场馆周边69个城中村，完成272个直管公房小区的整治改造，整顿309处街区户外广告，在100条重点大街开展创建“六无”示范活动。统一规划主要大街临街建筑墙体清洁工作。改造、新建标准公厕700座。

　　标本兼治，构建城市管理长效机制。按照职权法定、权责一致的原则，清理和规范行政执法主体，建立协调机制，落实责任制，继续推进城市管理综合行政执法。强化日常监管，夯实基层管理，综合治理黑车、游商等群众反映强烈的问题，下大力气排查“脏、乱、差、险”等管理盲点，维护管理好各类市政设施。从方便市民出发，做好各项公共服务设施的规划建设。广泛开展交通安全和法规宣传教育，培养市民遵守交通秩序的良好习惯。加强对流动人口的服务和管理。

　　各位代表，新的北京城市总体规划已经国务院原则通过。国务院的批复进一步明确了首都城市建设的指导思想、发展方向和总体部署，是指导首都建设与发展的纲领性文件。今年，要严格按照国务院批复和总体规划要求，完成教育、医疗等公共服务设施专项规划，组织编制中心城和新城控制性详细规划，加紧编制近期建设规划，明确建设时序和近期建设重点。从管理体制、机制上研究控制人口规模的方式和手段，着力提高人口素质。在市场配置资源的基础上，明确各区县的功能定位，探索制定新的评价指标体系，加强对各区县经济社会发展的宏观指导。强化规划实施监督，坚决维护城市规划的严肃性、权威性。

　　(二)切实抓好结构调整，加快推进经济增长方式转变

　　认真贯彻中央宏观调控的政策措施，保持固定资产投资的合理规模，增强消费对经济增长的拉动作用，着力解决经济发展中存在的重外延轻内涵、重增量轻存量、重数量轻质量、重速度轻效益、重引资轻服务等问题，进一步提高经济增长的质量和效益。

　　加快拥有自主知识产权的核心技术产业化步伐，提升高新技术产业和现代制造业发展水平。加大扶持力度，全面完成“五年上台阶”各项任务，进一步发挥中关村科技园区综合改革和创新示范的作用。完善智力要素参与分配的企业产权激励机制，抓紧解决中小科技企业融资难的问题。巩固软件和集成电路设计产业的国内领先地位，加快中关村软件园等产业基地建设。促进制造业集群化、集约化发展，加快北京经济技术开发区等重点产业基地建设，推动新增和搬迁改造的工业项目按产业类别和技术层次向开发区集中。抓好优势产业重点项目建设，促进汽车、电子等产业群和产业链的发展。制定并实施时装、食品、包装印刷行业发展纲要，扶持都市型工业的发展。

　　加快第三产业结构升级。抓住人民币业务对外资银行放开的机遇，加快商务中心区和金融街建设，积极吸引国内外各类金融机构及业务中心来京落户。推进地方性金融机构重组改革，在农村信用社基础上筹建市农村商业银行。按照支柱产业的目标要求，制定文化产业发展规划和支持政策，把文艺演出、出版发行和版权贸易、影视节目制作交易、动漫和互联网游戏研发制作、文化会展、古玩艺术品交易等优势行业做大、做强。积极推进国有文化企事业单位改革，鼓励社会资本兴办文化企业。促进高新技术产业和服务业融合，大力发展电子商务、信息增值服务、综合技术服务等新型服务业。全方位优化旅游环境，以高端产品带动旅游业、会展业快速发展。将影响交通和环境、存在重大安全隐患的小商品批发市场逐步调整出市中心区。加快连锁超市、便利店向郊区和新建社区延伸，鼓励和扶持社区服务业发展，满足城乡居民消费需求。

　　促进开放型经济发展。切实抓住并利用好当前有利的机遇，把扩大开放与加快优势产业发展、转变经济增长方式有机结合起来，吸引境内外资本向北京聚集，提高利用外资的质量。加快金融、文化、教育、旅游等领域对外开放，积极承接国际服务业的外包转移，促进服务贸易发展。充分发挥北京产权交易所的平台作用，制定相关政策，促进跨国公司以多种方式参与国有企业改组改造。完善和落实城市基础设施对外开放的政策措施，加快吸引外资步伐。从体制机制创新入手，依托重大工程项目，引进先进技术，提高消化、吸收和创新水平。对重点高新技术产品生产企业给予政策扶持和定期跟踪服务，提高产品技术含量和附加值，优化出口产品结构。建立健全以市和区县两级政府为主、相关中介机构配合的贸易促进工作机制，为中小企业开拓国际市场提供支持和帮助。创新对外经济合作方式，支持企业发展海外投资业务。

　　大力发展循环经济，建设节约型城市。坚持最严格的土地和水资源管理制度。完成全市土地利用总体规划修编，编制土地利用和供应计划，推行土地储备和开发，节约和集约用地，新上项目优先利用存量土地。加强用水总量控制和定额管理，充分运用水价机制，推动节水工作，万元生产总值水耗下降9%。建设亦庄节水示范区。加快推进平房区水、电一户一表改造。城区新建70公里再生水管线，推进园林绿化、道路降尘、景观用水、洗车等利用再生水工作，建设再生水灌区，全市再生水利用率达到30%。严格执行新的建筑节能标准，制定节约型居住区指标并开展试点工作。加大资金投入，加快推广资源节约利用新技术、新材料、新器具。加强宣传教育，增强全社会的节约意识。探索建立有利于循环经济发展的体制和规划，抓紧制定实施产业用地、水耗、能耗、环保、就业容量、带动效应等方面的标准，建立产业筛选和综合评价机制，使消耗高、污染大的产业和企业逐步退出。制定清洁生产审核强制性产品目录和实施细则，在医药等行业的典型企业做好试点示范工作。

　　加强经济运行调节。根据首都经济社会发展的能源需求，研究制定重点能源战略平衡规划，推进区域开发与合作。统筹协调能源结构调整与环境保护的关系，把握总量，优化结构，提高能源需求侧管理水平，完善应急预案，做好储备工作，缓解煤、电、油、气、运紧张状况，保证能源供应。搞好粮食、食用油等重要商品的储备、调运和供应调节，保障市场稳定。

　　精心编制好《北京市国民经济和社会发展第十一个五年规划》。继续做好第一次经济普查工作。积极参与国家京津冀地区战略规划的编制工作。

　　(三)稳定、完善和强化扶持政策，认真解决“三农”问题

　　切实保护农民利益。今年，各项支农措施的力度只能加大，已经给农民的实惠不能减少。坚持财政投入向郊区倾斜，做好现有各项政策的集成和落实，使政策发挥更大效应。全面完成农村土地承包经营权确权工作，推进农户土地承包经营权流转，促进经营主体多元化，发展适度规模经营。完成农村集体土地地籍调查，开展核发农村集体土地所有权证、农村集体建设用地使用权证的试点工作，研究制定农村集体建设用地使用权流转管理办法。按照“近郊区逐步推开，远郊区扩大试点”的思路，加快推进农村集体经济产权制度改革。

　　提高农业综合生产能力。实行最严格的耕地保护制度，确保基本农田总量不减少、质量不下降、用途不改变，落实到地块和农户。深化农业结构调整，全面落实“221行动计划”，努力构建农业“七大体系”，大力发展现代都市型农业。积极扶持龙头企业、专业合作组织和行业协会发展，提高农民的组织化程度，推进农业产业化经营。发展节水型农业，加大对小型农田水利基础设施建设的投入，开展对农民购买节水设备实行补助的试点，积极实行灌溉用水总量控制和定额管理，建设节水农田30万亩。按照强化公益性职能、放活经营性服务的要求，推进农业技术推广体系改革试点。积极探索建立政策性农业保险。加大农业生产投入品监管力度，推进食用农产品安全生产体系和农业标准化生产基地建设，实施农产品认证认可。做好重大动植物疫病的防控工作。

　　加大山区建设力度。把生态环境建设放在首位，继续做好植树造林、水源涵养和水土保持工作，切实搞好京津风沙源治理等防沙治沙工程，重点抓好荒山造林和小流域综合治理。完善扶持政策，促进特色林果业、绿色养殖业、休闲旅游业发展。制定综合开发配套政策，积极引导和鼓励社会资金投入山区建设。巩固和完善生态林补偿机制，进一步推动采煤、采矿业退出。完成7000名山区农民搬迁、安置任务，妥善处置搬迁后的山场和土地。

　　加快郊区工业化、城市化进程。加大政府投入，鼓励和引导各类社会资金进入郊区基础设施建设领域，重点向山区倾斜。完成30万农民饮用水达标硬件建设任务。新建村村通油路工程190公里。选择基础好的中心镇进行重点建设，促进人口集中、产业集聚。研究制定相关政策，积极推进旧村改造和新村建设试点，搞好土地整理，提高农村各类用地的利用率。引导加工制造业向郊区工业园区有序转移，加快区域优势产业和区域经济发展。完善和实施郊区旅游规划，大力扶持以旅游、休闲、度假为主的第三产业。探索建立信贷支持与财政支持的联动机制，扶持农民自主创业，加快郊区个体私营经济发展。

　　(四)深化体制改革，优化发展环境

　　推进国有企业和国有资产管理体制改革，促进非公有制经济发展。以“调整、改制、剥离、破产”为手段，加快调整国有经济布局和结构。以基础设施领域为重点，继续推进市属国有控股公司和企业集团重组整合。加快股份制改造步伐，促进混合所有制经济发展，力争年内完成国有企业全部改制任务的50%。基本完成国有大中型企业主辅分离、辅业改制任务。稳步实施扭亏无望企业的破产工作。完善国有资产监管经营体系，完成清产核资工作，规范企业负责人业绩考核和薪酬管理，全面实行国有资本收益收缴，扩大经营预算试点。开展董事会用人权改革试点，扩大企业负责人公开选聘范围。认真执行“非禁即入”政策，鼓励非公有制经济参与国有企业改组改造，进入城市基础设施、公用事业和社会事业领域，依法实现内资外资、国有民营平等待遇。进一步完善社会化服务体系和信用担保体系，建立中小企业发展资金，为具有发展潜力和示范作用的中小企业提供财政支持。

　　深化财政管理体制和投融资体制改革。坚持完善公共财政体制，逐步规范财政资金的供给范围，确保公益性基础教育、基础科研、计划生育、公共文化设施投入，继续加强社会保障体系、公共卫生体系和食品安全体系建设，切实保障社会公共安全和生产安全需求。完善市对区县财政体制，按照“财随事走、费随事转”的原则，明确两级政府的支出责任，实现事权和财权相统一。加大转移支付力度，完善转移支付资金分配办法。加强财政资金使用管理，在市级行政事业单位中全面推行国库集中支付，提高资金使用效益。强化税收征管，提高纳税服务水平。落实企业投资自主权，凡不使用政府投资的建设项目，一律不再实行审批制，政府仅对重大项目和限制类项目从维护社会公共利益角度进行核准，其他项目均改为备案制。进行经营性社会事业项目投融资改革试点，扩大公益性政府投资项目代建制范围，完善政府投资监督评价机制。政府投资的公益性项目，建设立项时必须同步测算运营成本。积极推行特许经营制度，全面推进新建基础设施项目建设运营的市场化，理顺公共产品和服务的价格，盘活基础设施存量资产，提高直接融资比重。增强政府投资的导向和带动作用，综合运用规划引导、土地供应等政策手段，优化投资的产业和区域结构。

　　推进城市管理体制改革。按照理顺街道办事处与政府专业管理部门关系、理顺社区民主自治与政府行政管理关系的改革思路，积极开展试点工作，认真总结经验，健全街道办事处统筹和监督机制，把专业管理和服务的责任落到实处。加强社区建设，完善公民参与机制，增强社区民主自治功能。积极培育社区服务组织，充分发挥社区服务中心的作用，不断提高服务质量和水平。进一步规范物业管理，探索正确处理社区居委会、业主委员会和物业管理单位关系的有效措施。

　　进一步改善投资软环境。把转变政府职能作为优化发展环境的首要任务，加快制定完善各领域投资的相关规则和标准，探索运用政府指导、行政合同等间接管理、动态管理手段，进一步完善行政投诉机制，提高行政效率和服务水平。加大政务公开力度，向社会公布行政许可事项的许可依据、条件、程序、期限和相对人的权利义务，完善规范性文件制定、公布制度，拓展政府规章和规范性文件自由索取范围。加快电子政务建设，扩大网上办公范围，推进政府部门资源共享。实行部门联动，狠抓政策的集成与落实，切实降低企业投资运行成本。分类研究招商引资中的遗留问题，逐项加以解决。进一步完善企业信用服务体系，研究制定社会信用体系建设方案。加强行政投诉中心的组织协调和监管作用，严厉查处违法行政行为。以打击盗版和商标、专利侵权为重点，开展保护知识产权专项行动，有效遏制各种侵犯知识产权的行为。

　　(五)大力发展各项社会事业，切实加强精神文明建设

　　加快社会事业发展，同样要贯彻落实科学发展观。加大结构调整力度，继续把投入重点向郊区和基层倾斜，逐步缓解城乡、区域之间社会事业发展的不均衡状况。坚决杜绝不考虑成本和需求的盲目投入，实现投入方式由粗放型向集约型转变。通过重组、置换等多种形式，调整盘活存量资源，提高有效利用程度。

　　全面实施首都教育发展战略。认真落实各项人才政策，大力开发人力资源，通过各种形式的教育和培训，不断提高劳动者素质。继续加大教育投入，加快教育结构调整，切实提高教育质量。优先发展农村义务教育，改善农村中小学办学条件，完成100所中小学布局调整，为500所中小学配备体育设施。推行基础教育课程改革，扎实推进素质教育。加强教师队伍建设，重点抓好中小学校长和农村教师培训。加快职业教育改革和发展，大力培养应用型、技能型人才，重点支持50个紧缺专业和50个农村远程教育示范站点建设。充分发挥中央在京高等教育资源优势，调整市属高校发展定位，优化学科专业结构。积极推进教育体制改革，支持和规范民办教育健康发展。加大教育乱收费治理力度，认真搞好校园安全和周边环境整顿。

　　增强科技创新能力。深化科技体制改革，建立以企业为主导、产学研相结合的创新机制，促进企业成为技术创新主体。完善鼓励创新的财税、金融、用地、人才等政策，支持企业加大研发投入，提高自主创新能力，开发具有自主知识产权的核心技术。扩大技术市场的平台作用和交易规模，推动科技资源与实业投资、金融资本紧密结合，促进科技资源优势转化为产业优势。在信息通讯、生物医药、新材料、光机电一体化、现代农业等领域，支持一批关键、共性技术的研究开发，提升重点行业整体水平与核心竞争力。通过组织实施一批项目，加快科技创新资源向郊区的辐射，发挥科技在城乡统筹发展中的支撑作用。

　　加快医疗卫生事业发展。制定卫生区域发展规划，加强卫生资源的结构调整和优化配置，完善卫生服务体系。继续以卫生基础设施建设和人才培养为重点，落实城市卫生资源支农措施，完成覆盖山区的1486个社区卫生服务站的基本建设。加强公共卫生基础设施建设，完善首都地区急救网络，重点推进社区卫生服务中心急救点建设，完成乡镇急救点建设规划。加快推进传染病专科接诊室、专用门诊建设，抓好地坛医院搬迁和佑安医院扩建等重点项目。积极推进医院投融资管理体制改革，推动国有医院产权制度改革试点，探索建立医院国有资产监管机构，逐步实现管办分开。巩固医院纠风工作成果，加大对收受回扣、“红包”、“开单提成”和乱收费等问题的治理力度。进一步规范药品集中招标采购工作，努力降低群众医药费用负担。大力开展健康教育，重点普及防治艾滋病知识。

　　提高文化事业发展水平。深入开展群众文化活动，构建以基层为重点的公共文化服务体系，支持48个农村文化中心、759个农村文化室建设，完成深山区260个自然村广播电视“村村通”，开展民族民间文化保护试点。加快首都博物馆、中国电影博物馆等文化重点工程建设。积极探索运用市场化手段繁荣公益性文化事业的新途径。搞好群众体育基础设施建设与管理，广泛开展全民健身活动，增强群众身体素质。继续实施奥运夺金计划，提高竞技体育水平。

　　全力推进精神文明建设。软环境建设是筹办奥运最具挑战性的任务。要认真落实人文奥运行动计划实施意见，深入开展首都文明礼仪宣传教育实践活动，启动奥运会志愿者活动计划，加强历史文化遗产保护，扩大对外文化交流，继续搞好市民讲外语活动，普及奥运比赛项目相关知识，提高全体市民的文明素质。广泛开展精神文明创建活动，倡导科学、健康、文明的生活方式，全面推进文明社区、文明村镇、文明行业建设，争创全国文明城市。搞好普法宣传教育，积极开展法律援助。繁荣哲学社会科学事业，普及科学知识。抓好未成年人思想道德建设，构建具有首都特色的学校、家庭、社会相结合的教育体系。坚持不懈地开展“扫黄”“打非”斗争，建立文化市场综合执法机构，强化文化市场和互联网管理。加强国防后备力量建设，深入开展国防教育，增强全社会国防意识，支持驻京部队建设，做好双拥共建工作。研究制定加强民族工作的措施，努力促进少数民族乡村发展。认真贯彻国务院《宗教事务条例》，进一步落实宗教房产政策。继续推进京港、京澳、京台交流与合作，做好侨务工作。加强人口和计划生育工作。依法保障妇女、未成年人、老年人和残疾人的合法权益，加快无障碍设施建设。

　　(六)坚持以人为本，努力构建社会主义和谐社会的首善之区

　　积极探索统筹城乡就业的新机制，不断增加城乡居民收入。新增城镇就业19万人，城镇登记失业人员再就业17万人。加大对中小企业和个人创业的信贷支持，全面推广经济建设与促进就业“三同时”制度。继续实施“三年三十万”社区就业岗位开发计划，增强岗位开发的针对性。继续推进就业倍增计划，实现1000名失业人员成功创业，带动1万人就业的目标。健全农村富余劳动力就业登记服务制度，将有劳动能力和就业愿望的人员纳入全市统一的就业服务体系，研究制定转移就业办法，加强乡镇和村级就业服务组织建设，加大职业指导、职业介绍和技能培训力度。研究制定特殊帮扶政策，引导失业人员跨地区就业，缓解失业率较高地区的就业压力。继续采取政策倾斜、承诺服务、就业助理等措施，为就业困难人员提供一对一全程跟踪服务。大力推行集体合同制度，规范企业用工行为。加大对建筑企业工资支付情况的监控力度，基本解决拖欠农民工工资问题。

　　坚持不懈地做好社会保障工作。加快推进建设征地转非劳动力、整建制农转居人员、来京务工人员参加社会保险，尽快将他们纳入覆盖范围。制定农村社会保险制度建设的指导性意见，积极推进农村养老保险制度改革。完善新型农村合作医疗相关政策，扩大农民受益面，逐步提高保障水平。继续完善城镇养老保险制度，开展做实基本养老保险个人账户工作，积极建立企业年金制度。以私营、个体和灵活就业人员为重点，扩大社会保险覆盖面，确保各项基金征缴率在95%以上。

　　大力推进城乡社会救助体系建设，切实保障困难群众基本生活。完善最低生活保障制度，建立规范的低保标准调整机制，依据居民消费品价格指数变化情况，适时采取临时补贴等应急措施，强化动态管理，保障低保人员基本生活。不断完善专项救助制度，切实发挥综合解困效应。全面落实城乡医疗救助制度，资助所有农村低保对象参加当地合作医疗。继续实施农村特困户危房改造计划，扩大城市住房救助范围。研究城镇供热救助办法。加大临时救助力度，努力解决高于低保标准的低收入家庭生活困难问题。做好农村低收入群体增收的动态监测工作，有针对性地进行扶持和帮助。

　　切实解决群众住房困难。以确保市民生命安全为切入点，坚持政府主导、群众参与的原则，发挥市场机制作用，完善法规政策，采取多种方式加快危旧房改造步伐。听取专家意见，保护古都风貌，疏散旧城过度密集的人口。加大财政扶持力度，加快配套基础设施建设，稳步推进6个历史文化保护区试点项目。重点做好解危排险工作，解决8000户居民的居住安全隐患问题。竣工200万平方米经济适用住房，抓好廉租房建设和筹集工作，优先满足危旧房改造的需要。严格执行拆迁程序和政策，依法文明拆迁。围绕直接关系群众生活的热点、难点问题，办好55件重要实事。

　　全力做好首都安全工作。认真清查全市危险源分布情况，排查消除各类安全事故隐患，对短期内确实难以解决的隐患，要严防死守，加快整治。严格执行各项法律、法规和制度，落实社会单位主体责任和政府监管责任，积极推进安全质量标准化，规范安全生产行为，加强日常监管，从源头上防范各类安全事故发生。建立全市统一的食品安全监控系统、农副产品质量安全定期抽检公布制度，加大药品、保健食品、医疗器械、化妆品检查监测力度，确保食品、药品安全。强化产品质量监督检查和专项整治，每季度向社会公布重点商品检测结果，积极引导安全消费。进一步完善应急体系，建立健全应对各类突发事件的管理体制和工作机制，整合应急资源，提高安全工作专业队伍水平，认真组织好演练，增强反应和处置能力。加强消防基础设施建设。在全市普及预防各类灾害知识，提高市民的安全意识和自我防范救助能力。

　　坚决维护首都稳定。正确处理新形势下人民内部矛盾，畅通和拓宽信访等民愿诉求渠道，建立信访反馈与政策实施研究联动机制，积极预防和妥善处理群体性事件。完善人民调解、治安行政调解、司法调解相衔接的工作制度，形成各部门协调配合的工作机制和工作格局，促进社会和谐稳定。进一步强化社会治安综合治理，以创建平安社区、平安村为载体，积极探索治安防范工作的社会化、科技化、法制化途径，提高全社会的治安防控能力。依法打击各类犯罪活动，加强对黄赌毒的查禁力度，重视反恐斗争，增强群众的安全感，保护人民生命财产安全。

　　三、加强政府自身建设，提高依法行政能力

　　面对首都改革开放和现代化建设的新形势、新任务、新要求，各级政府必须牢固树立科学发展观和正确政绩观，按照科学执政、民主执政、依法执政对政府的要求，加快职能转变，加强基础工作，不断提高行政效能和服务水平。

　　第一，全面推进依法行政，建设法治政府。坚持依法治市，认真贯彻行政许可法，落实《全面推进依法行政实施意见》，更新政府行政理念，完善行政程序，规范行政行为，健全行政监督机制，把依法行政贯穿于政府工作的方方面面，努力做到合法行政、程序正当、高效便民、诚实守信、权责统一。加强行政立法，重点完成食品安全、重大公共活动安全管理、城市基础设施特许经营等方面的立法任务，提高政府立法质量。健全公众参与、专家论证和政府决定相结合的科学民主决策机制，完善行政决策程序，建立决策跟踪反馈和责任追究制度。深化行政执法体制改革，继续推行行政执法责任制、评议考核制，建立行政执法人员责任追究制度，促进行政机关和工作人员切实履行职责。坚决贯彻市人大及其常委会决议，坚持重大事项报告制度，认真做好市政府组成人员述职工作，自觉接受监督。坚持重大问题决策前认真听取政协及各民主党派、工商联、无党派人士的意见，自觉接受民主监督。认真办理人大议案、建议和政协提案，以点带面推动各方面工作。进一步加强行政机关的内部监督，自觉接受司法监督和社会监督。

　　第二，加强基础工作，提高行政水平。做好基础工作是提高行政能力的前提。各级政府、各部门，特别是领导干部要认真分析存在的问题和薄弱环节，周密制定整改措施和实施方案，通过不懈努力，使各级政府、各部门的基础工作得到明显加强，决策的科学性和行政效能得到明显提高。具体要求是：全面深入掌握本级政府、本部门的职能责任，明晰干部的岗位职责，严格落实责任制，做到“职责清”；对工作领域的历史过程、工作现状、面临矛盾以及问题成因，能够心中有数，做到“情况明”；建立健全以准确可靠数据为基础的数字化管理机制，基础数据全面完整，动态变化及时更新，部门之间信息资源共享，做到“数字准”；加强政治理论学习和业务知识学习，全面掌握并正确执行相关法律、法规和政策，实现“素质高”。同时，要敢于提出和认真遵循较高的工作标准和工作要求，力争使各项工作走在全国的前列。广大基层公务员是推动首都各项事业发展的重要力量，要进一步加强全市基层公务员队伍建设，关心他们的学习和生活，支持他们做好工作。

　　第三，以“保持共产党员先进性教育活动”为契机，进一步搞好政风建设。按照中央和市委的部署，在行政机关集中开展好先进性教育活动，认真解决在思想、组织、作风以及工作方面存在的突出问题，使人民政府始终成为人民利益的忠实维护者，使广大公务员树立正确的理想信念和高尚的道德情操，永葆人民公仆的本色。坚持实事求是，严格按照客观规律办事，努力使各项工作经得起实践检验；坚持群众路线，认真听取群众意见，自觉接受群众监督，充分调动人民群众的积极性和创造性，发挥全体市民在首都现代化建设中的主体作用；坚持求真务实，说实话、办实事、求实效，力戒浮躁和推诿扯皮，把更多的时间和精力用在调查研究和工作落实上；坚持勤俭节约，反对铺张浪费，坚决制止不切实际、不计成本、不问效果的错误做法；坚持勤政廉政，充分发挥行政监察、审计等职能部门的作用，坚决纠正行业不正之风和以权谋私，进一步加强廉政建设和反腐败斗争，真正做到“为民、务实、清廉”。

　　各位代表，首都改革开放和现代化建设正处于关键时期，我们深知任务非常艰巨，责任十分重大，使命无比神圣，前景无限美好。让我们紧密团结在以胡锦涛同志为总书记的党中央周围，以邓小平理论和“三个代表”重要思想为指导，全面落实科学发展观，坚定信心，迎难而上，开拓进取，扎实工作，为实现“新北京、新奥运”战略构想而努力奋斗！
